# The Story of a Ship Journey, Malaria, and the *HBB* Gene IVS-II-745 Mutation: Circassian Immigration to Cyprus

**DOI:** 10.1055/s-0041-1726336

**Published:** 2021-03-16

**Authors:** Mahmut C. Ergoren, Sehime G. Temel, Gamze Mocan, Munis Dundar

**Affiliations:** 1 Department of Medical Genetics, Faculty of Medicine, Near East University, Nicosia, Cyprus; 2 Rare Diseases Research Group, DESAM Institute, Near East University, Nicosia, Cyprus; 3 Department of Medical Genetics, Faculty of Medicine, Bursa Uludag University, Bursa, Turkey; 4 Department of Medical Pathology, Faculty of Medicine, Near East University, Nicosia, Cyprus; 5 Department of Medical Genetics, Faculty of Medicine, Erciyes University, Kayseri, Turkey

**Keywords:** Circassians, β-thalassemia, IVS-II-745, Cyprus, malaria, genetic fitness

## Abstract

**Background** During 19th century, the Circassians were secluded from their lands and forced to migrate to Ottoman Empire properties. Approximately 2,346 Circassians were exiled from Istanbul to Cyprus Island. During the deportation journey, many of Circassian passed away in consequence of malaria and unknown reasons. Overall, 1,351 survivor Circassians managed to reach the island, however, many of them had faced with endemic malaria again in Cyprus. An autosomal recessive hematological disorder thalassemia was the second endemic health condition after malaria, whereas thalassemia carriers show resistance to malaria infections.

**Materials and Methods** A large Cypriot family with 57 members whose grandparents were supposed to be in that ship journey has been investigated in this study. Polymerase chain reaction (PCR)–amplification refractory mutation system (ARMS) analysis technique was used for genotyping the *HHB* gene.

**Results** The *human β-globin* ( *HBB* ) gene c.316–106C > G (IVS-II-745) (II-745) heterozygous variation have been detected.

**Conclusion** Overall, this study is a very good example for a typical natural selection. In this case, one single gene point mutation did not limit survival in the society; natively, it increased their survival changes to form new colonization and the inheritance of the mutation to the next generations.

## Introduction

During the 19th century, the autochthonous Circassians (Adyghe, Cherkess) of the Caucasus, who resisted to dominate the Eurasian Basin, were driven away from the mainland as a consequence of “The Great Game” between the British Empire, the Ottoman Empire, and the Russian Tsarist. All Circassian tribes, particularly near the coastal zone or around Krasnaya Polyana (Red Plateau) region, were relentlessly deported in view of the geopolitical anxiety of Russia. The number of Circassians directly killed by the Russians was estimated to be more than 500,000. 1 According to the British war historian Allen, the number of Circassians placed in the Ottoman territory was more than 600,000 between the years of 1863 and 1864. 2

Immigration to Istanbul was banned due to epidemic diseases; therefore, shipment was directed to the Balkans. The Circassians, who had settled to Cyprus, were originally from Samsun, prior to Caucasus and Istanbul. Approximately 2,346 Circassians were exiled from Istanbul to Cyprus by three Ottoman flagged Aufdromachi vessels named “Revan-i Ticaret,” “Hıfz-i Rahman,” and “Eflak.” During the deportation journey, many people could not survive due to malaria and unknown reasons. 3 It was suspected that they fell victim to malaria or typhus. In fact, only 1,351 of 2,346 Circassians arrived to Cyprus.

An autosomal recessive hematological disorder, β-thalassemia, was one of the most serious endemic health conditions in Mediterranean region after malaria. 4 5 6 During 1944 to 1946 Dr. Alan Fawdry was first to report thalassemia in Cyprus 5 and the control of malaria was successful in Cyprus between the years of 1946 and 1950. 7 Haldane suggested that heterozygous carriers for β-thalassemia are less predispose to severe malarial infections, since cells containing reduce or absent synthesis of globin chains are not very conductive to malarial parasite expansion properly within the erythrocytes. 8 Previous studies have revealed that five mutations were detected amongst the Turkish Cypriots 9 10 and Greek Cypriots. 11 The results were generally similar IVS-I-110 (G→A) 74.1%, IVS-I-1 (G→A) 7.3%, IVS-I-6 (T→C) 7.8%, IVS-II-745 (C→G) 6.5%, codon 39 (C→T) 0.9%, unknown 3.4% were the frequency of Turkish Cypriots and IVS-I-110 (G→A) 79.0%, IVS-I-6 (G→A) 6.3%, IVS-I-I (T→C) 6.0%, IVS-II-745 (C→G) 4.1%, codon 39 (C→T) 1.8%, and the other 3.4% were the frequency for Greek Cypriots, respectively ( Table 1 ). 10 11 The mutation denoted as IVS-II-745 (C→G) was introduced to the Eastern Mediterranean region during Muslim groups movement living in former Turkish territories to Southern Europe as a consequence of the Ottoman Empire falling (starting from 1914 AD), consequently contributing to a racial admixture. 12 13 In addition, the IVS-II-745 (C→G) mutation was detected in less heterogenic regions, for instance Northern, Southern, and Western Anatolia 14 15 16 ( Table 2 ). In this study, we aimed to trace the introducer of the mutation in the island and explained natural selection process.

**Table 1:** Frequency and regional distribution of IVS-II-745 C > G β-thalassemia mutation in Cyprus

Larnaca/Famagusta region	Limassol region	Nicosia region	Paphos region	North Cyprus	Overall
4.7%	5.3%	4.0%	2.0%	6.5%	4.5%

**Table 2:** Frequency and regional distribution of IVS-II-745 C > G β-thalassemia mutation across the world

Country	Frequency (%)
Syria	16.6
Jordan	14.2
Egypt	7.2
North Cyprus	6.5
Greece	6.3
Italy	5.0
Turkey	5.0
Germany	4.3
Cyprus	4.1
Morocco	4.0
Lebanon	4.0
Macedonia	3.9
Bulgaria	3.7
Iran	3.7
Czech Republic	3.5
Slovakia	2.8
Tunisia	7.5
Israel	2.5
Spain	1.7
United Kingdom	1.7
Azerbaijan	0.8
Argentina	0.7
Portugal	0.4
Sri Lanka	0.2
India	0.04

## Materials and Methods

### Patients and Genotyping

Venous blood was collected with ethylenediaminetetra-acetic acid (EDTA) tubes from 57 Turkish Cypriot family members and genomic DNA was isolated using PureLink Genomic DNA Mini Kit (Thermo Fisher Scientific, Waltham, Massachusetts, United States) for sequencing. Medical history was questioned and written informed consent form obtained from all the patients. DNA extraction was conducted under a class-II laminar flow using autoclaved pipets to minimize the risk of contamination in a class-II laminar flow hood. All solutions and equipments were ultraviolet (UV) treated to prevent any potential contamination. Mutation screening was performed in the *human β-globin* *(HBB)* gene using polymerase chain reaction (PCR)–amplification refractory mutation system (ARMS) analysis for point mutations as described by Sozuöz et al. 10 Informed consents were obtained from all participants. This study has been approved by the institutional ethics committee (registration number: YDU/2020/77–978).

## Results

### Studied Patients and Genotyping

Fifty-seven members of the conserved Circassian family members whose grandparents were driven away from their mainland and migrated to Cyprus in early 1860s was screened for common thalassemia-causing mutations using PCR-ARMS. The sequence analysis revealed heterozygous IVS-II-745 (C→G) mutation in 12 family members (II:1, II:3, II:9, II:11, III:1, III:3, III-5, III:20, IV:1, IV:4, IV:5, IV:16; Fig. 1 ).

**Fig. 1:**
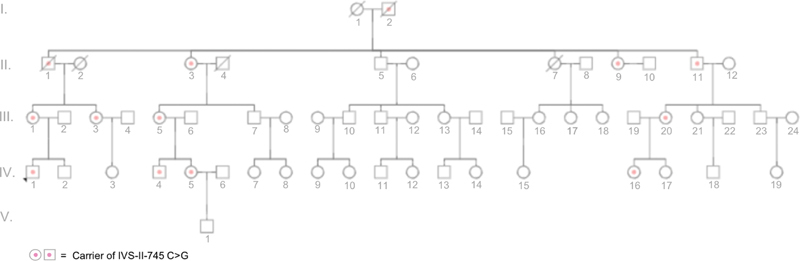
Pedigree of the Cerkez family. Affected family members by a heterozygous IVS-II-745 (C→G) are shown in the pedigree.

## Discussion

The family data can be used to summarize that this studied family has relations to Caucasia, it further demonstrates that ancestors of the family were at an advantage during the migration to Cyprus, as this mutation protected them from malaria. The example provided in this study represents a very example for natural selection scenario. Overall, this current study indicated that the *HBB* gene IVS-II-745 (C→G) did not limit survival, in fact it enhanced the survival changes of the migrating family members, allowing them to form new colonization, and inheritance of the mutation to their offspring.

## Conclusions

1. The *HBB* gene IVS-II-745 (C→G) mutation has been studied in a larger family.
2. This study involves a standard population genetics topic including migration, gene flow, natural selection, and adaptation.
3. This study contributes both to the fields of history and human genetics.
